# Super-late pulmonary recurrence after radical esophagectomy for esophageal squamous cell carcinoma

**DOI:** 10.1016/j.ijscr.2020.05.068

**Published:** 2020-06-06

**Authors:** Akira Umemura, Yuji Akiyama, Takeshi Iwaya, Keisuke Koeda, Ryo Sugimoto, Tamotsu Sugai, Fumitaka Endo, Shigeaki Baba, Haruka Nikai, Hiroyuki Nitta, Takeshi Takahara, Koki Otuska, Toshimoto Kimura, Hajime Saito, Hiroyuki Deguchi, Makoto Tomoyasu, Akira Sasaki

**Affiliations:** a Department of Surgery, Iwate Medical University, Yahaba 028-3695, Japan; b Department of Molecular Diagnostic Pathology, Iwate Medical University, Yahaba 028-3695, Japan; c Department of Thoracic Surgery, Iwate Medical University, Yahaba 028-3695, Japan

**Keywords:** Esophaegal cancer, Pulmonary metastasis, Late recurrence, Oligometastasis, Squamous cell carcinoma

## Abstract

**Introduction:**

Pulmonary metastases from esophageal squamous cell carcinoma (ESCC) are often detected bilateral and multiple lesions and are often accompanied by metastases to other sites. The concept of oligometastasis has been developed, and limited distant metastases have been considered as indications for surgical resection for the purpose of extending overall survival. We herein present a long-surviving case of super-late pulmonary recurrence of ESCC, seven years after radical esophagectomy.

**Presentation of case:**

A 71-year-old woman who underwent radical subtotal esophagectomy with three-field lymph node dissection with a diagnosis of an advanced poorly differentiated ESCC with cT3N1M0 seven years ago visited our hospital. Chest X-ray and computed tomography at the 7-year follow-up revealed a solitary pulmonary tumor, 1.5 cm in diameter, at the right middle lobe without any extrapulmonary metastases; however, we could not diagnose whether primary lung cancer or pulmonary metastasis of ESCC was present. Therefore, we performed thoracoscopic partial resection of the right middle lobe. A histopathological examination including immunohistochemical staining revealed that the tumor was not derived from both alveolar epithelium and neuroendocrine cells and was diagnosed as pulmonary oligometastasis of ESCC. She has been followed for four years without re-recurrence.

**Conclusion:**

Pulmonary oligometastases of ESCC should be considered as surgical indications if the tumor is detected after a long disease-free interval without any extrapulmonary recurrences.

## Highlights

• Super-late pulmonary recurrence of esophageal squamous cell carcinoma.
• Successfully resection and long survive after pulmonary metastasectomy.
• Surgical indication of pulmonary oligometastasis of esophageal cancer.

## 1 Introduction

Esophageal cancer (EC) is notorious for its early spread and poor prognosis after radical esophagectomy. Tumor recurrence, the main cause of death in patients with EC after treatment, has been reported to occur in more than half of patients even after extended operation with three regional lymphadenectomies [1]. The great majority of recurrence develops within three years after radical esophagectomy, not only at the local site but also in distant organs. In contrast, recurrences manifesting more than five years after radical esophagectomy are extremely rare; hence, little is investigated about the clinical and pathological features of such cases.

Pulmonary metastases from EC are often detected bilateral and multiple lesions, and are often accompanied by metastases to other sites. Therefore, only a few of these cases will be candidates for pulmonary resection [2].

Here, we have reported a long-surviving case of super-late pulmonary recurrence after radical esophagectomy for esophageal squamous cell carcinoma (ESCC), with some considerations about the therapeutic strategy for pulmonary recurrence of ESCC.

This work has been reported in line with the SCARE criteria [3].

## 2 Case presentation

**Fig. 1:**
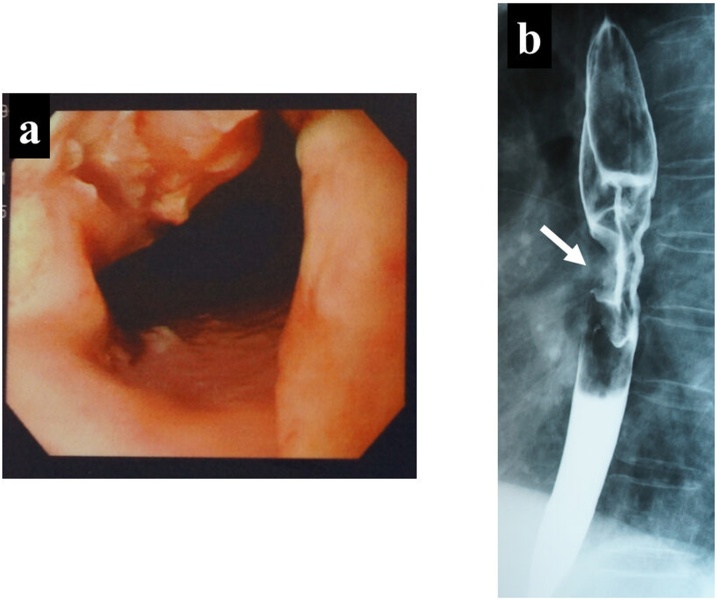
a: An esophagogastroduodenoscopy revealed a type-3 tumor at the middle intrathoracic esophagus. b: A barium esophagography also revealed an irregular stricture of the middle intrathoracic esophagus (white arrow).

**Fig. 2:**
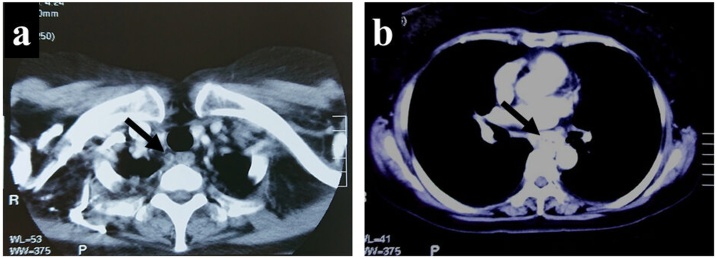
a, b: A CT scan revealed the upper mediastinal lymph node swelling and thickening of the esophageal wall.

A 71-year-old woman presented with a 2-month history of dysphagia and chest pain after swallowing and came to our hospital with a complaint of worsening symptoms. An esophagogastroduodenoscopy revealed a type-3 tumor at the middle intrathoracic esophagus (Fig. 1a), and a barium esophagography revealed an irregular stricture of the middle intrathoracic esophagus, measuring about 5 cm in length (Fig. 1b). Endoscopic biopsy specimens taken from the tumor histologically showed poorly differentiated squamous carcinoma, and an enhanced computed tomography (CT) work-up revealed thickening of the esophageal wall and swelling of a mediastinal lymph node without any distant organ metastases (Fig. 2a, b). She was diagnosed with an advanced ESCC with cT3N1M0, and underwent radical subtotal esophagectomy with three-field lymph node dissection.

**Fig. 3:**
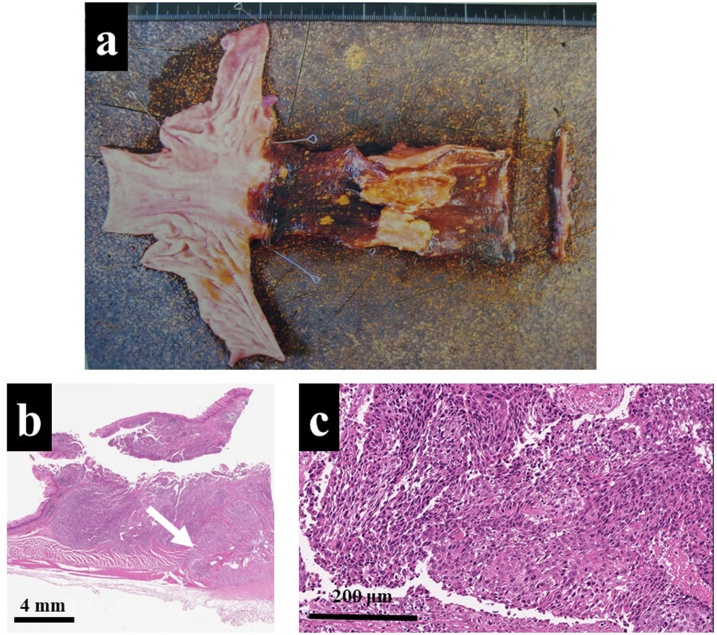
a: The gross examination of the specimen showed a tumor measuring 45 × 15 mm in consonance with unstained area of iodine staining. b: The tumor invaded into the adventitia without exposure of the tumor (white arrow) (HE-stain, × 10). c: A high-power field revealed the tumor cells were poorly differeciated squamous cell carcinoma (HE-stain, × 40).

**Fig. 4:**
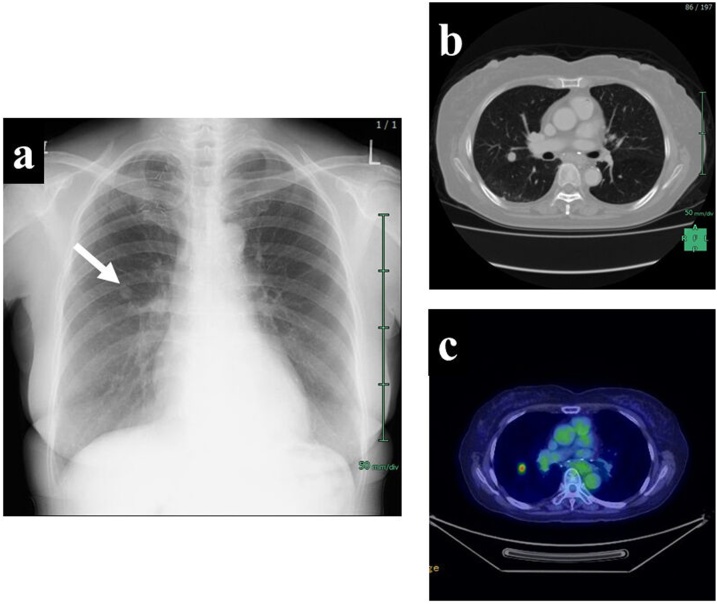
a: A chest X-ray at 7-year follow-up revealed a solitary pulomonary tumor at right lung (white arrow). b: A CT scan revealed the tumor with 1.5 cm of diameter. c: A PET-CT also showed the pulmonary tumor had abnormal uptake with 4.9 of standardized uptake value maximum.

The gross examination of the specimen showed that there was a type-3 tumor measuring 45 × 15 mm in consonance with an unstained area of iodine staining (Fig. 3a). Microscopic findings of the low-power field revealed invasion into the adventitia without exposure of the tumor (Fig. 3b), and those of high-power field also revealed poorly differentiated squamous cell carcinoma with multiple lymph node metastases; these findings led to a diagnosis of pT3N1M0 (Fig. 3c). However, she had been sequentially administered adjuvant doublet chemotherapy using cisplatin and 5-FU, grade 4 hematological toxicity, and hepatobiliary disorders aborted it during the first course. She was then followed for 6 years without recurrence; however, chest X-ray and CT examinations at the 7-year follow-up revealed a 1.5-cm-diameter solitary pulmonary tumor at right middle lobe with (Fig. 4a, b). In addition, positron emission tomography CT (PET-CT) also showed abnormal uptake of this pulmonary tumor (Fig. 4c). Although we could not preoperatively perform CT-guided percutaneous lung biopsy due to the deep location of the tumor, any regional lymph nodes or distant metastases were undetected; therefore, we performed thoracoscopic partial resection of the right middle lobe.

**Fig. 5:**
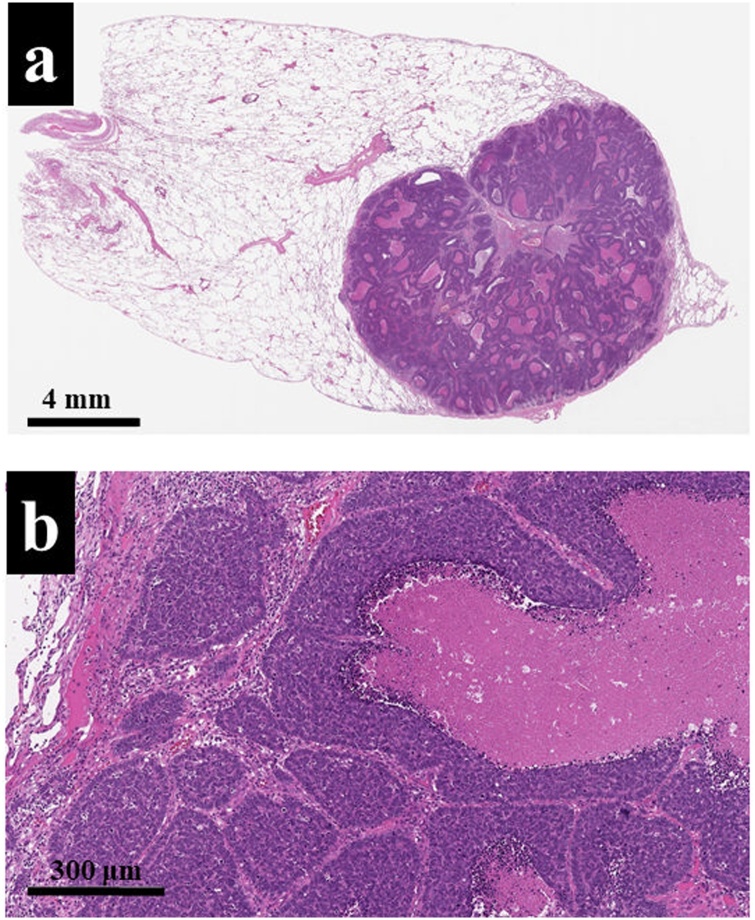
a: A low-power field revealed that there was a well-demarcated tumor with necrotizing compartments (HE-stain, × 10). b: A high-power field also revealed that the tumor cells proliferated without having basement membrane-like structure (HE-stain, × 40).

**Fig. 6:**
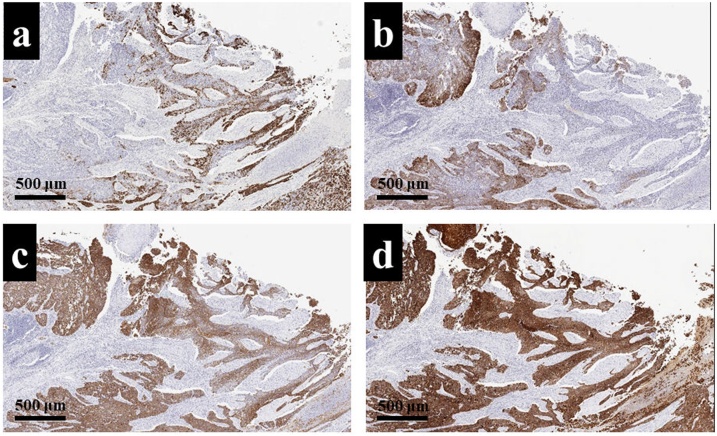
An immunohistochemical examination revealed that the tumor cells of primary ESCC were positive for CK14 (a), CK18 (b), CK19 (c), and CK5/6 (d) (× 10).

**Fig. 7:**
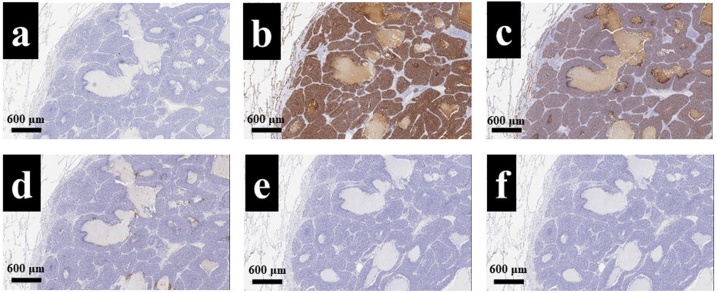
An immunohistochemical examination of the lung tumor also revealed that the tumor cells were negative for CK14 (a), napsin A (e), and synaptophysin (f). On the other hand, the tumor cells were strongly positive for CK18 (b) and CK19 (c), focally positive for CK5/6 (d) (× 10).

Microscopic findings of low-power field revealed that there was a solid tumor with necrotizing compartments, and those of high-power field also revealed that tumor cells proliferate without having a basement membrane-like structure (Fig. 5a, b). From these findings, morphological features led to the diagnosis of poorly differentiated squamous cell carcinoma. An immunohistochemical examination was performed to identify the origin of the tumor primary lung cancer or pulmonary metastasis from ESCC. The tumor cells of primary ESCC were positive for CK14, CK18, CK19, and CK5/6 (Fig. 6a–d). On the other hand, the tumor cells of the lung tumor were negative for CK14, napsin A, and synaptophysin (Fig. 7a–f). From these immunohistochemical features, the pulmonary tumor was not derived from both alveolar epithelium and neuroendocrine cells; thus, we finally made the diagnosis of super-late pulmonary recurrence of ESCC. This patient was discharged from the hospital on postoperative day 10 without any complications, and no recurrence was noted for four years after pulmonary metastasectomy.

## 3 Discussion

The most common site of distant metastasis in patients with EC is the lung. Furthermore, patients with EC are at high risk for metachronous primary lung cancer due to their habit for smoking. Therefore, secondary pulmonary malignancies are a major problem after definitive treatment of EC [4]. However, distinguishing pulmonary metastasis from primary lung cancer in patients with a treatment history of ESCC is very difficult owing to the similarities in radiographic and histopathological appearance [5].

Usually, treatment strategies for primary lung cancer differ from those of pulmonary metastases. Distant metastasis is considered to be a systemic disease; therefore, the administration of systemic therapies rather than local therapies is favored. However, the reported results of chemotherapy for pulmonary metastases from EC were poor, with few complete response cases and short survival times [6]. From this background, surgeons made great efforts to discover positive results of pulmonary metastasectomy; currently, pulmonary metastasectomy of limited metastases of soft tissue sarcoma, renal cell carcinoma, germ cell tumors, and osteosarcoma have the potential to extend overall survival time compared to systemic chemotherapy and other local therapies. In response to these clinical achievements, a concept of oligometastasis has been developed [7], and various distant metastases of malignant tumors characterized by fewer than 5 metastases have been considered as indications for surgical resection [8]. In this case, the patient had only one pulmonary tumor without any distant metastases, and she was not tolerant of standard chemotherapy; therefore, we regarded it as oligometastasis, which should be resected.

Currently, there is no consensus or guidelines on the management of distant oligometastasis of ESCC. Pulmonary metastases are commonly observed in patients with a surgical history of EC; therefore, the accumulation of case series has proven the efficacy of pulmonary metastasectomy for oligometastasis from ESCC. When we decide to perform pulmonary metastasectomy of ESCC, we have to consider the factors affecting survival time. According to previous retrospective case series, short disease-free interval (DFI) less than 13–16 months, prior extrapulmonary recurrence, poor differentiation of ESCC, and multiple pulmonary metastases were estimated as unfavorable prognostic factors [2,5,6,9]. These reports also showed that the 5-year survival rates were 29.6–43.5% in patients without unfavorable prognostic factors [9,10]. Kanamori et al. reported that a 3-year survival rate with multiple pulmonary metastasis was 0% compared with 56.2% of patients with solitary pulmonary metastasis [5]. The present case was free from most unfavorable factors without poor differentiation, and with a very long DFI and a solitary tumor, we therefore performed pulmonary metastasectomy.

The surgical benefits of resection of pulmonary oligometastases are described above; however, the re-recurrence rate was reported as 22–70% after pulmonary metastasectomy [5,11]. From these results, a closed follow-up is needed to detect re-recurrence and to avoid missing the opportunity for radical treatment and adjuvant chemotherapy after resection of pulmonary oligometastases, which should be considered to extend overall survival. Fortunately, this case has already lived without re-recurrence for four years.

## 4 Conclusion

We have reported a long-surviving case of super-late pulmonary recurrence of ESCC seven years after radical esophagectomy. Pulmonary oligometastases of ESCC should be considered as surgical indications if the tumor is detected after long DFI without extrapulmonary recurrence. However, relatively high re-recurrence rates have been reported after metastasectomy; therefore, closed follow-up is needed and further powered analyses are warranted to clarify the true benefit of pulmonary metastasectomy of ESCC.

## Conflicts of interest

The authors have no conflicts of interest.

## Funding

The case report has no sponsors.

## Ethical approval

This case report is exempt from ethical approval by our institution.

## Consent

Written informed consent was obtained from the patient for publication of this case report and accompanying images. A copy of the written consent is available for review by the Editor-in-Chief of this journal on request.

## Author contribution

Umemura Akira – study concept, data collection, draft preparation.

Akiyama Yuji, Iwaya Takeshi, Koeda Keisuke, Endo Fumitaka, Baba Shigeaki, Nikai Haruka – surgical therapy for this patient and follow-up.

Sugimoto Ryo, Sugai Tamotsu – histopathological diagnosis.

Saito Hajime, Deguchi Hiroyuki, Tomoyasu Manabu – pulmonary resection for this patient.

Nitta Hiroyuki, Takahara Takeshi, Koki Otuka, Toshimoto Kimura, Sasaki Akira – data interpretation.

## Registration of research studies

Not necessary in this case report.

## Guarantor

Akira Umemura.

## Provenance and peer review

Not commissioned, externally peer-reviewed.
